# Analysis of Deregulated microRNAs and Their Target Genes in Gastric Cancer

**DOI:** 10.1371/journal.pone.0132327

**Published:** 2015-07-14

**Authors:** Simonas Juzėnas, Violeta Saltenienė, Juozas Kupcinskas, Alexander Link, Gediminas Kiudelis, Laimas Jonaitis, Sonata Jarmalaite, Limas Kupcinskas, Peter Malfertheiner, Jurgita Skieceviciene

**Affiliations:** 1 Institute for Digestive Research, Lithuanian University of Health Sciences, Kaunas, Lithuania; 2 Department of Gastroenterology, Lithuanian University of Health Sciences, Kaunas, Lithuania; 3 Department of Gastroenterology, Hepatology and Infectious Diseases, Otto von Guericke University, Magdeburg, Germany; 4 Division of Human Genome Research Centre, Faculty of Natural Sciences, Vilnius University, Vilnius, Lithuania; University of Pennsylvania School of Medicine, UNITED STATES

## Abstract

**Background:**

MicroRNAs (miRNAs) are widely studied non-coding RNAs that modulate gene expression. MiRNAs are deregulated in different tumors including gastric cancer (GC) and have potential diagnostic and prognostic implications. The aim of our study was to determine miRNA profile in GC tissues, followed by evaluation of deregulated miRNAs in plasma of GC patients. Using available databases and bioinformatics methods we also aimed to evaluate potential target genes of confirmed differentially expressed miRNA and validate these findings in GC tissues.

**Methods:**

The study included 51 GC patients and 51 controls. Initially, we screened miRNA expression profile in 13 tissue samples of GC and 12 normal gastric tissues with TaqMan low density array (TLDA). In the second stage, differentially expressed miRNAs were validated in a replication cohort using qRT-PCR in tissue and plasma samples. Subsequently, we analyzed potential target genes of deregulated miRNAs using bioinformatics approach, determined their expression in GC tissues and performed correlation analysis with targeting miRNAs.

**Results:**

Profiling with TLDA revealed 15 deregulated miRNAs in GC tissues compared to normal gastric mucosa. Replication analysis confirmed that miR-148a-3p, miR-204-5p, miR-223-3p and miR-375 were consistently deregulated in GC tissues. Analysis of GC patients’ plasma samples showed significant down-regulation of miR-148a-3p, miR-375 and up-regulation of miR-223-3p compared to healthy subjects. Further, using bioinformatic tools we identified targets of replicated miRNAs and performed disease-associated gene enrichment analysis. Ultimately, we evaluated potential target gene *BCL2* and *DNMT3B* expression by qRT-PCR in GC tissue, which correlated with targeting miRNA expression.

**Conclusions:**

Our study revealed miRNA profile in GC tissues and showed that miR-148a-3p, miR-223-3p and miR-375 are deregulated in GC plasma samples, but these circulating miRNAs showed relatively weak diagnostic performance as sole biomarkers. Target gene analysis demonstrated that *BCL2* and *DNMT3B* expression in GC tissue correlated with their targeting miRNA expression.

## Introduction

The discovery of microRNAs (MiRNAs) and establishment of their role in molecular pathways has brought a huge advance in molecular biology [1][2]. These small non-coding RNAs comprised of ~22bp are involved in post-transcriptional regulation of gene expression. MiRNAs are characterized by high stability in biological samples making these molecules an attractive target in biomarker research field [3][4]. Accumulating evidence shows that miRNAs are involved in major carcinogenesis pathways [5]. Previous studies have revealed that deregulation of miRNAs occurs virtually in all major types of cancer [5][6]. Furthermore, miRNAs have been shown to have a diagnostic or prognostic role and even potential clinical implications for targeted gene therapy in cancer patients [7][8].

The incidence of gastric cancer (GC) in Western countries has declined over the last decades; however, this type of cancer still accounts for nearly one million of new disease cases worldwide and carries a very high mortality rate [9]. Recent research on GC has revealed many new insights into the pathogenesis of this malignancy. Nevertheless, the exact mechanisms of malignant transformation from *Helicobacter pylori* (*H*. *pylori*) infection to chronic atrophic gastritis, intestinal metaplasia and GC is still poorly understood [10]. Current hypothesis of GC development involves combined effects of bacterial, host and nutritional factors; however, to date, this theory suggests very few clinically sound translational implications [11]. The majority of GC cases are diagnosed in late stages of the disease, which is associated with poor patient outcomes. Therefore, one of the major focuses in GC research is the evaluation of potential molecular biomarkers that could be used for early non-invasive diagnostics of this malignancy.

The crucial role of miRNAs in GC has been shown in different studies [12]. Volinia et al. have provided one of the first miRNA expression profiles in GC tissue showing a specific deregulation pattern [13]. Further studies have also reported significant deregulation of miRNAs belonging to miR-17, miR-21, miR-223, miR-135 and many other families. Among reported studies in GC tissues, miR-21, miR-25, miR-92, miR-223 were the most consistently up-regulated miRNAs, while miR-375 and miR-148 were the most consistently down-regulated [14][15][16]. Most of these studies have looked at miRNA profile in patients with GC and paired adjacent normal gastric mucosa [14]. Important studies have shown that miRNAs are already deregulated in early stages of gastric carcinogenesis including *H*. *pylori* gastritis and premalignant stages of gastric atrophy and intestinal metaplasia [17][18]. Interestingly, some of miRNAs have opposite deregulation directions in the presence of GC, as separate profiling studies show significant inconsistency among deregulated miRNAs [14]. MiR-9 was found to be up-regulated in two studies [15][19], while two other papers showed significant down-regulation of this miRNA in GC tissues [13][20]. These discrepancies regarding opposite deregulation results for miR-9 and many other miRNAs are most likely linked to differences in anatomical location of the GC, histological subtype, disease stage, profiling platforms, statistical analysis and many other potential confounding factors. Besides, the majority of currently published studies on miRNA profile in GC tissues cover subjects from Asian countries; meanwhile, data on European GC patients is still scarce [21].

The goal of our present study was to determine miRNA profile in GC tissues and compare it to the normal healthy gastric mucosa using wide coverage miRNA TaqMan low density arrays. In further analysis, we aimed to validate our profiling results in tissue and plasma of a larger group of GC patients and healthy controls of European descent. Ultimately, we evaluated the potential target genes of confirmed differentially expressed miRNA and performed disease-association gene enrichment analysis of their target genes.

## Materials and Methods

### Ethics statement

The study was approved by the Kaunas Regional Biomedical Research Ethics Committee (Protocol No BE-2-10). All patients signed an informed consent form to participate in the study.

### Study population

Tissue specimens were prospectively collected between 2011 and 2014 in Departments of Gastroenterology and Surgery, Hospital of Lithuanian University of Health Sciences (Kaunas, Lithuania). The study included a total of 51 control subjects and 51 GC patients, which were divided into the profiling (GC, n = 13; controls, n = 12) and validation cohorts (GC, n = 38; controls, n = 39). All subjects were of European descent. Gastric biopsy samples were obtained from antral part of the stomach from control subjects who were referred for upper GI endoscopy due to dyspeptic symptoms and had no previous history of malignancy. GC tissue specimens were obtained from surgical specimens immediately after resection from patients undergoing primary surgery for GC with no preoperative irradiation and chemotherapy. Gastric adenocarcinoma in GC patients was verified by histology and classified according to Lauren into diffuse and intestinal types [22]. *H*. *pylori* status was assessed in GC and control subjects using indirect ELISA to detect the serum-specific IgG antigen (Virion/Serion GmbH, Wünrzburg, Germany). Clinical and pathological characteristics of the patient cohorts including age, gender and disease stage are summarized in **Table 1.**

**Table 1:** Clinical characteristics of the gastric cancer patients and healthy controls.

		Profiling cohort (n = 25)			Validation cohort (n = 77)		
		cancer (n = 13)	normal (n = 12)	p value	cancer (n = 38)	normal (n = 39)	p value
Age	Mean ± SD	71 ± 13.5	59.2 ± 11.5	p = 0.0269	67.36 ± 12.19	55.69 ± 16.1	p = 0.0006
Gender	Male, n (%)	9 (69.23)	5 (41.66)		25 (65.79)	15 (38.46)	
	Female, n (%)	4 (30.77)	7 (58.34)	p = 0.1654	13 (34.21)	24 (61.54)	p = 0.02
Lauren classification	Diffuse, n (%)	3 (23.08)	-	-	14 (36.84)	-	-
	Intestinal, n (%)	10 (76.92)	-	-	20 (52.63)	-	-
	Mixed, n (%)	-	-	-	1 (2.63)	-	-
	Unknown, n (%)	-	-	-	3 (7.89)	-	p = 0.4
*H*.*pylori* infection	Positive, n (%)	8 (61.54)	-		17 (44.74)	13 (33.34)	
	Negative, n (%)	4 (30.77)	12 (100)		6 (15.79)	18 (46.15)	
	Unknown, n (%)	1 (7.69)	-	p = 0.0015	15 (39.47)	8 (20.51)	p = 0.0124
Tumor localization	Cardia, n (%)	2 (15.38)	-	-	7 (18.42)	-	-
	Corpus, n (%)	7 (53.85)	-	-	14 (36.84)	-	-
	Antrum, n (%)	3 (23.08)	-	-	13 (34.21)	-	-
	Linitis plastica, n (%)	1 (7.69)	-	-	2 (5.26)	-	p = 0.76
TNM staging	I, n (%)	-		-	2 (5.26)	-	-
	II, n (%)	1 (7.69)	-	-	5 (13.16)	-	-
	III, n (%)	7 (53.85)	-	-	17 (44.74)	-	-
	IV, n (%)	3 (23.08)	-	-	14 (36.84)	-	-
	Unknown, n (%)	2 (15.38)	-	-	-	-	p = 0.11
T	1/2	1 (7.69)	-	-	9 (23.68)	-	
	3	7 (53.85)	-	-	13 (34.21)	-	-
	4	3 (23.08)	-	-	15 (39.47)	-	-
	Unknown, n (%)	2 (15.38)	-	-	1 (2.63)	-	p = 0.13
N	0	2 (15.38)	-	-	13 (34.21)	-	-
	1	7 (53.85)	-	-	6 (15.79)	-	-
	2	1 (7.69)	-	-	7 (18.42)	-	-
	3	1 (7.69)	-	-	9 (23.68)	-	-
	Unknown, n (%)	2 (15.38)	-	-	3 (7.89)	-	p = 0.06-
M	0	10 (76.92)	-	-	10 (26.32)	-	-
	1	3 (23.08)	-	-	14 (36.84)	-	-
	Unknown, n (%)	-	-	-	14 (36.84)	-	p = 0.003
Diferentiation grade	1/2	2 (15.38)	-	-	14 (36.84)	-	-
	3	8 (61.54)	-	-	23 (60.53)	-	-
	Unknown, n (%)	3 (23.08)	-	-	1 (2.63)	-	p = 0.04

*H*. *pylori—Helicobacter pylori;* GC–gastric cancer.

### Tissue sample preparation and RNA extraction

Gastric tissue samples were stored in RNAlater (Ambion, Austin, TX, USA) at +4°C and 24 hours later stored at -80°C. 30 mg of tissue was homogenized in sterile condition before total RNA isolation with mirVana miRNA Isolation Kit (Ambion, Austin, TX, USA) for profiling study and miRNeasy Mini Kit (Qiagen, Valencia, CA, USA) for validation study, according to the manufacturers’ instruction. The quality of RNA was assessed using the Nanodrop 2000 spectrophotometer (Thermo Scientific, USA). Qualitative examination of RNA integrity was performed by electrophoresis on agarose gel (5%).

### Plasma sample preparation and RNA extraction

Plasma samples were collected from health patients and patients with GC. The miRNA expression in plasma cohort consisted of plasma samples from 38 gastric cancer patients and 39 healthy volunteers. Venous blood was collected in EDTA anticoagulation vacuum tubes and was centrifuged at 3500 rpm for 15 min at room temperature; separated plasma was transferred into 1.5 ml tubes and placed in a −80°C freezer for short-term storage. Small RNAs were extracted from 200 μl of plasma using the miRNeasy Serum/ Plasma Kit (Qiagen, Valencia, CA, USA) as per the manufacturer’s instructions.

### MiRNA profiling using the TaqMan Low-Density Array

MiRNA profiling was performed with the TaqMan Array Human MiRNA Card A v2.1 which enabled to quantify 377 human miRNAs cataloged in miRBase v20 [23]. Briefly, 350 ng of total RNA was initially reverse transcribed using the Megaplex RT set pool A version 2.1 (Applied Biosystems, Carlsbad, California, USA) and 800 μl of cDNA product was then loaded on the TaqMan Array Human MiRNA Card and run on the ViiA 7 Real-Time PCR System (Applied Biosystems). Primary analysis was done using ViiA 7 Software (Applied Biosystems) to get expression in terms of Ct. Data analysis was performed using Bioconductor HTqPCR package [24]. MiRNAs with a Ct value > 37 were considered unamplified. MiRNAs which were not amplified in more than 25% of samples were considered to be lowly expressed and, therefore, excluded from further analysis. MiRNA expression data was normalized using rank invariant normalization. NormFinder and geNorm algorithms were used to identify a reference gene from TLDA data for validation study [25]. According to the algorithms miR-127-3p showed lowest Ct variance and was chosen as a reference gene for the validation study. Recent studies show that the expression levels of RNU6A and some other small nuclear RNAs might be unstable in certain tissues and conditions and may not serve as best reference genes [26][27][28][29]. One recent publication has also identified miR-127-3p as a good candidate for reference gene selection [30].

### Quantitative reverse transcription polymerase chain reactions (qRT-PCR)

Reverse transcription (RT) reactions were performed using TaqMan MiRNA Reverse Transcription Kit (Applied Biosystems, Carlsbad, California, USA) and miRNA-specific RT stem-loop primers (Applied Biosystems, Carlsbad, California, USA). Singleplex reactions were carried out in a volume of 7.5 μL. Each reaction comprised 2.08 μL nuclease free water, 0.74 μL 10× RT buffer, 0.08 μL dNTPs (100 mM), 1.5 μL 5× miRNA-specific RT primers, 0.1 μL RNase inhibitor, 0.5 μL Multiscribe Reverse Transcriptase and a fixed volume of miRNA template (2,5 μL). RT was carried out in a thermocycler under the following conditions: 16°C for 30 min, 42°C for 45 min and 85°C for 5 min, followed by a hold at 4°C.

TaqMan real-time PCR reactions were performed in duplicate reactions comprising 6.25 μL TaqMan 2× Universal PCR Master Mix with No AmpErase UNG (Applied Biosystems, Carlsbad, California, USA) 0.625 μL 20× miRNA-specific primer and probe mix of the TaqMan MiRNA Assay Kit (Applied Biosystems, Carlsbad, California, USA), 3 μL of the reverse transcription product and 2.625 μL nuclease free water. RT-PCR was carried out by using a 7500 Fast Real-Time PCR System (Applied Biosystems, Carlsbad, California, USA) under the following conditions: 95°C for 10 min, then 40 cycles of 95°C for 15 s, 60°C for 60 s, followed by a hold at 4°C. Each sample was run in duplicate. The data were analyzed with automatic settings for assigning the baseline, and average Ct and SD values were calculated. The expression level of miRNA in the tissue was normalized to miR-127-3p and the expression level in plasma was normalized to hsa-miR-16-5p. The results were calculated using the ΔΔCT method [31]. The data were analyzed with automatic settings for assigning the baseline, and average Ct and SD values were calculated.

cDNA for *BCL2* and *DNMT3B* gene expression analysis was synthesized from 500 ng of RNA using a High-Capacity cDNA Reverse Transcription Kit (Applied Biosystems, Carlsbad, California, USA). Singleplex reactions were carried out in a volume of 7,5 μL. Each reaction comprised 2.1 μL nuclease free water, 1 μL 10× RT buffer, 0,4 μL dNTPs (100 mM), 1 μL 10× RT Random primers, 0.5 μL Multiscribe Reverse Transcriptase and a fixed volume of miRNA template (2,5 μL). RT-PCR was carried out in a thermocycler under the following conditions: 25°C for 10 min, 37°C for 120 min and 85°C for 5 min, followed by a hold at 4°C. Before qPCR cDNA samples were diluted 1:1 with nuclease free water and 2 μl used in 10,5 μl RT-qPCR reactions together with 6,25 μl 2x TaqMan Fast Universal Master mix (Life Technologies, Carlsbad, California, USA), 0.625 μl 20x TaqMan Gene Expression Assay (Life Technologies, Carlsbad, California, USA) and 3.625 μl sterile water. RT-qPCR assays were run in triplicate on a 7900HT Fast Real-Time PCR System (Applied Biosystems, Carlsbad, California, USA) under the following conditions: 95°C for 10 min, then 40 cycles of 95°C for 15 s, 60°C for 60 s, followed by a hold at 4°C. The expression levels of *BCL2* and *DNMT3B* in the tissue was normalized to *ACTB*.

### Target gene network analysis

The lists of validated target genes of the candidate miRNAs were obtained from miRTarbase v4.5 (mirtarbase.mbc.nctu.edu.tw) and miRecords v4.0 (mirecords.biolead.org) databases. Gene-disease association data were retrieved from the DisGeNET database (http://www.disgenet.org/). In order to identify GC-associated genes, the term “gastric adenocarcinoma” (umls: C0278701) was used as query for the database. Biological networks were created using Cytoscape v3.2 open-source software with CyTargetLinker application [32].

### Statistical analysis

The clinical characteristics among groups were compared using the *χ*2 test and Fisher’s exact test for qualitative data, and t-test for quantitative data. The TLDA data was analyzed using t-test and Benjamini Hochberg correction for false discovery rate such that differential expression was considered to be significant with a p < 0.01. The data was normalized using rank invariant normalization [33] and analyzed using the HTqPCR package. The validation and plasma qPCR expression data was analyzed using nonparametric Mann–Whitney U-test. A Benjamini Hochberg adjusted p < 0.05 was considered to be statistically significant. For qRT-PCR data, the relative expression levels of each target miRNA (Log2 relative level) were calculated according to the difference in CT values between the target miRNAs and miR-127-3p in tissue samples and miR-16-5p in plasma samples (ΔCT) [34]. Hypergeometric test was used for GC-associated target enrichment analysis. A receiver operating characteristic (ROC) curve was generated for each miRNA using ΔCT data. The area under curve (AUC) value and 95% confidence intervals (CI) were calculated to determine the specificity and sensitivity. To analyze diagnostic value of combined changes in plasma miRNA levels, the univariate gene expression average algorithm was used [35]. ROC curves were generated using ROCR package [36]. All data analyses were performed using R version 3.1.1 software.

## Results

### MiRNA expression profiling of GC and healthy gastric mucosa tissue by TLDA

**Fig 1:**
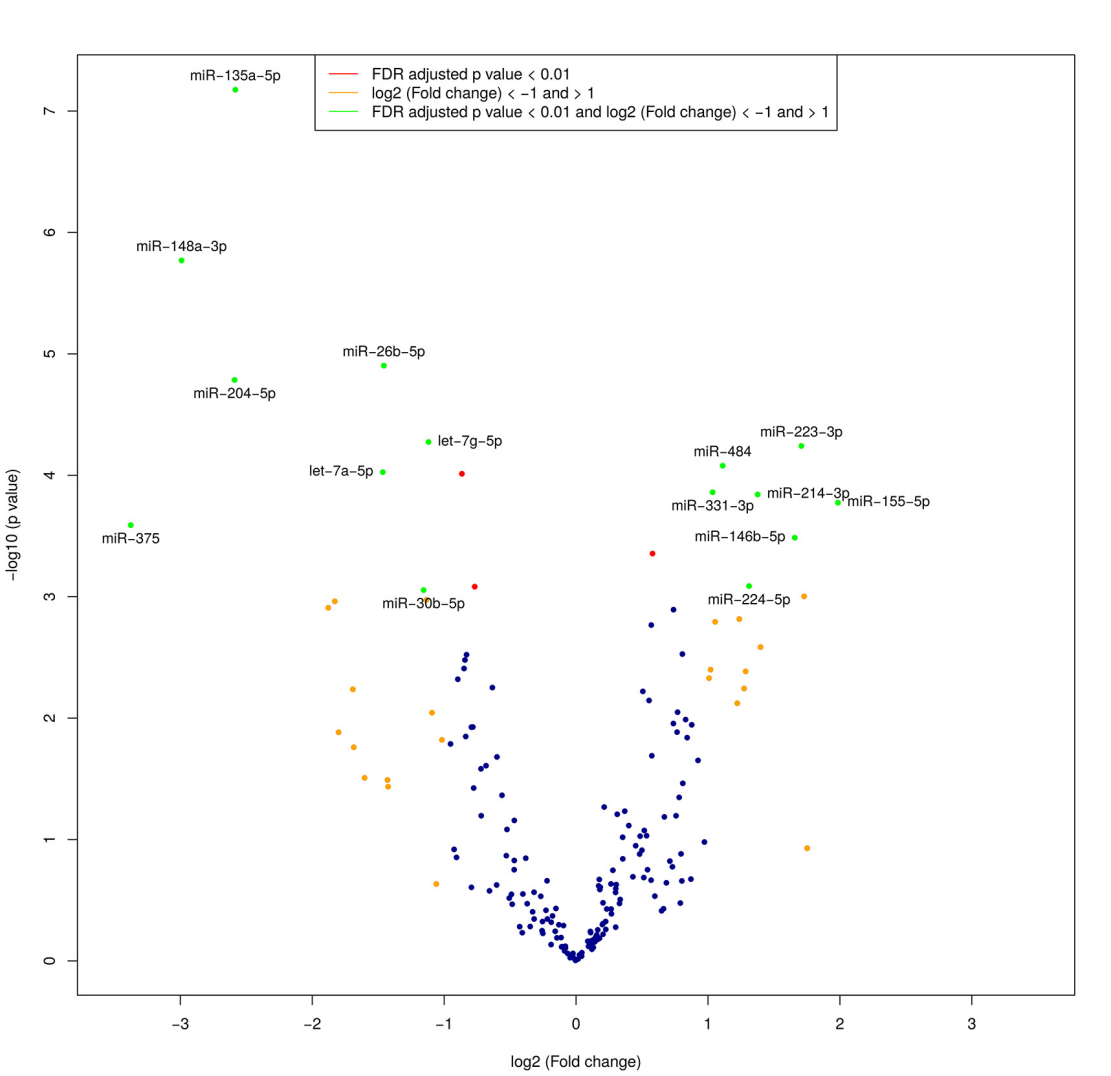
The volcano plot of aberrantly expressed miRNAs detected in TLDA. The green color represents significantly (FDR adjusted p < 0.01) differentially expressed miRNAs with fold change > 2. The red color indicates significantly differentially expressed miRNAs with fold change < 2.

**Fig 2:**
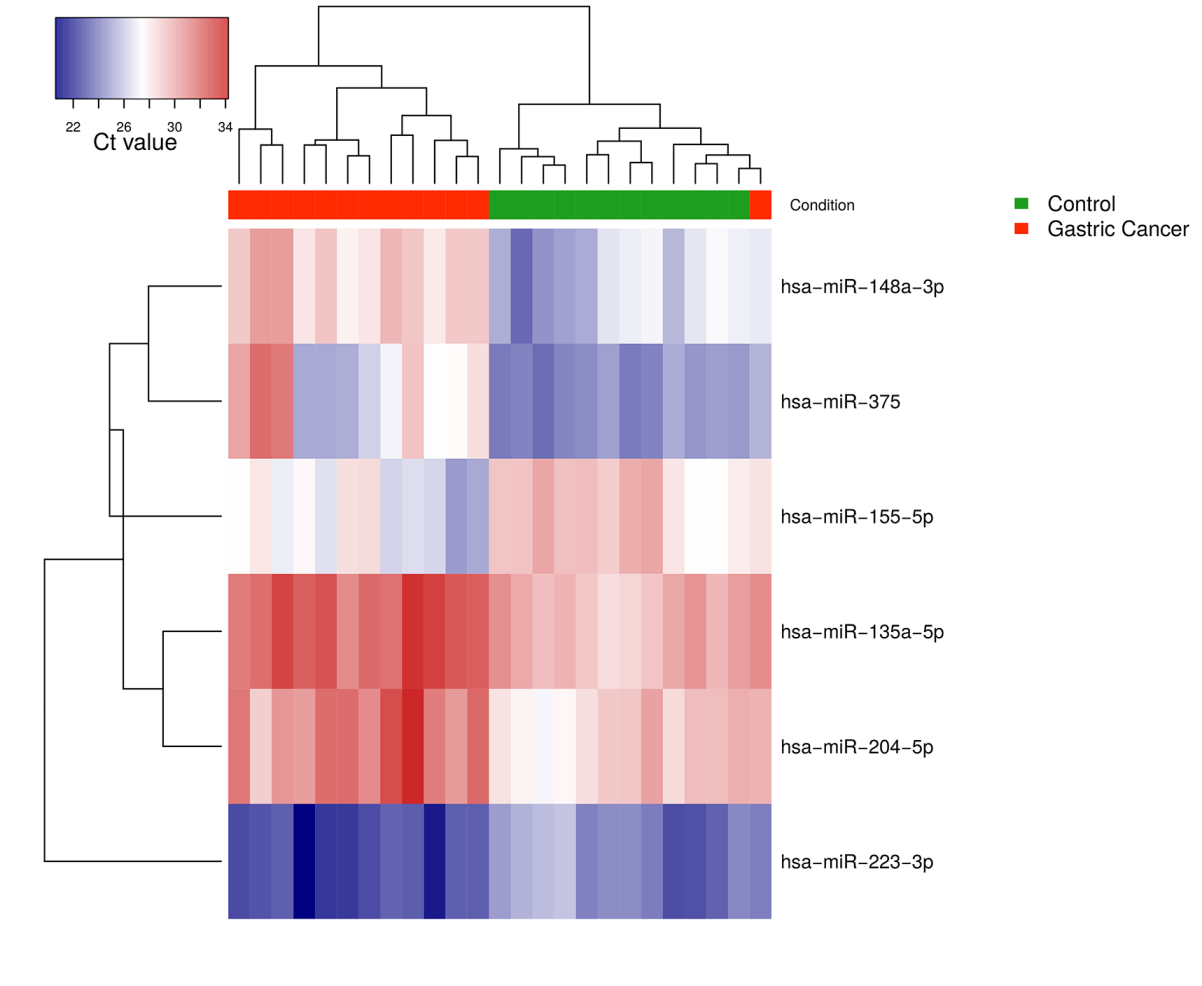
Heat-map diagram of a two-way hierarchical clustering analysis consisting of the 6 most differentially expressed miRNAs in GC tissue and normal gastric mucosa. The red and blue colors indicate expression level of miRNAs in terms of normalized Ct value. Upper color labeling shows GC patient samples in red and controls in green. Distance of hierarchical clustering was measured using the Euclidean method.

TaqMan Human miRNA Low-Density Array analysis was performed to identify candidate miRNAs exhibiting altered expression in gastric cancer tissue. Tissue miRNA profiles from GC patients were compared with profiles of gastric mucosa from healthy individuals. After filtering for low abundant miRNAs (Ct value > 37 and non-detectable in over 25% of samples), a set of 193 miRNAs (from total 377) remained for further analysis. A comparison of cancerous versus normal tissue identified 15 differentially expressed miRNAs, 7 of which were up-regulated and 8 were down-regulated with FDR adjusted p-value <0.01 and fold change >2 (**Table 2**). Results are demonstrated in volcano plot (**Fig 1**). Among these, 6 miRNAs (miR-135a-5p, miR-148a-3p, miR-204-5p, miR-375, miR-223-3p and miR-155-5p), showing the highest significance and fold change values, were selected for the validation study. Hierarchical clustering under Euclidean distance of selected miRNA normalized expression data revealed two subgroups: one corresponding to the control group and the second corresponding principally to the patient group. (**Fig 2**).

**Table 2:** List of deregulated miRNAs determined by TaqMan Human miRNA Low-Density Array between gastric cancer (n = 13) and normal gastric (n = 12) tissues (FDR adjusted p-value <0.01 and fold change >2).

Genes	FoldChange	log2 (Fold Change)	mean of control Ct	mean of case Ct	p-value	FDR adjusted p-value
Down-regulated						
hsa-let-7a-5p	0.362	-1.465	26.969	28.434	9.42E-005	0.002085
hsa-let-7g-5p	0.461	-1.118	26.182	27.300	5.32E-005	0.001844
hsa-miR-135a-5p	0.167	-2.583	29.725	32.307	6.67E-008	0.000013
hsa-miR-148a-3p	0.126	-2.991	25.904	28.894	1.70E-006	0.000164
hsa-miR-204-5p	0.166	-2.589	28.783	31.372	1.64E-005	0.000790
hsa-miR-26b-5p	0.364	-1.457	24.940	26.397	1.25E-005	0.000790
hsa-miR-30b-5p	0.449	-1.155	24.743	25.898	0.000882	0.009462
hsa-miR-375	0.096	-3.376	24.378	27.753	0.000257	0.003821
Up-regulated						
hsa-miR-146b-5p	3.156	1.658	24.302	22.643	0.000327	0.004504
hsa-miR-155-5p	3.959	1.985	28.993	27.008	0.000168	0.002703
hsa-miR-214-3p	2.596	1.376	28.410	27.033	0.000144	0.002525
hsa-miR-223-3p	3.268	1.708	26.777	25.069	5.73E-005	0.001844
hsa-miR-224-5p	2.482	1.311	32.817	31.506	0.000818	0.009392
hsa-miR-331-3p	2.050	1.035	27.299	26.264	0.000138	0.002525
hsa-miR-484	2.160	1.111	25.790	24.678	8.34E-005	0.002085

### MiRNA validation in GC and healthy gastric mucosa tissue with qRT-PCR

**Fig 3:**
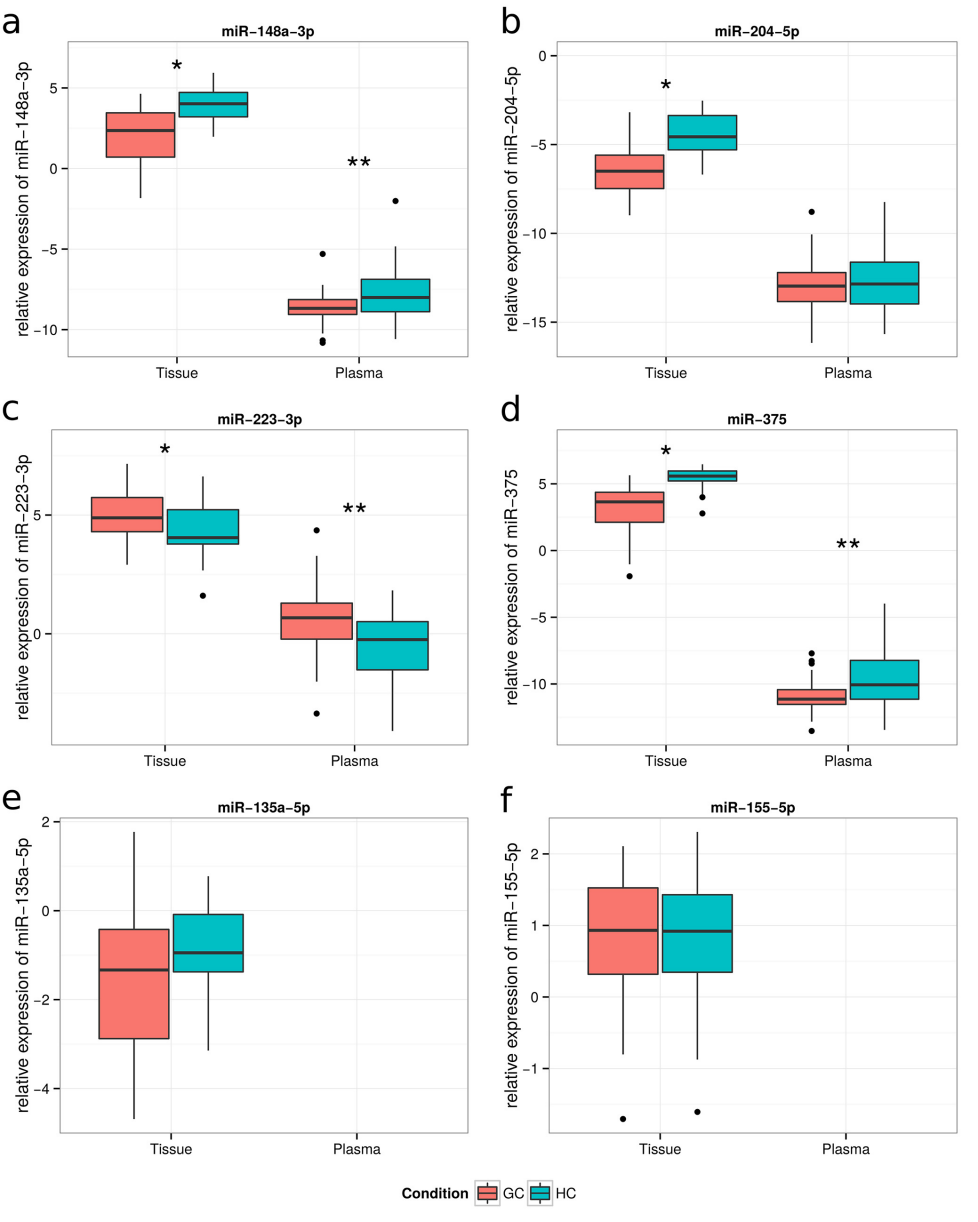
Expression levels of candidate miRNA in tissue and plasma samples. The boxplots for miR-148a-3p (**a**); miR-204-5p (**b**); miR-223-3p (**c**); miR-375 (**d**); miR-135a-5p (**e**) and miR-155-5p (**f**) represent the results of qRT-PCR comparing GC samples with healthy controls. qRT-PCR data are represented as log2 2-(deltaCt) values. The red and blue colors shows expression levels of candidate miRNAs in tissue and plasma samples, respectively. *—FDR adjusted p < 0.05 by Mann-Whitney U test in tissue. **- FDR adjusted p < 0.05 by Mann-Whitney U test in plasma.

The six candidate miRNAs selected for verification were quantified using qRT-PCR. For reference gene selection the TLDA data was analyzed using two different algorithms (NormFinder and geNorm) to identify reference genes. MiR-127-3p showed highest expression stability across all of the samples in TLDA data and because of this feature miR-127-3p was chosen as a reference gene to normalize qPCR data. The expression levels of miR-148a-3p, miR-204-5p, miR-223-3p and miR-375 showed significant differential expression in the same direction as in the discovery study (FDR adjusted p < 0.05), while no significant differences were detected in the expression levels of miR-135a-5p and miR-155-5p (FDR adjusted p > 0.05; **Fig 3**).

### Plasma miRNAs as potential biomarkers for gastric cancer

**Fig 4:**
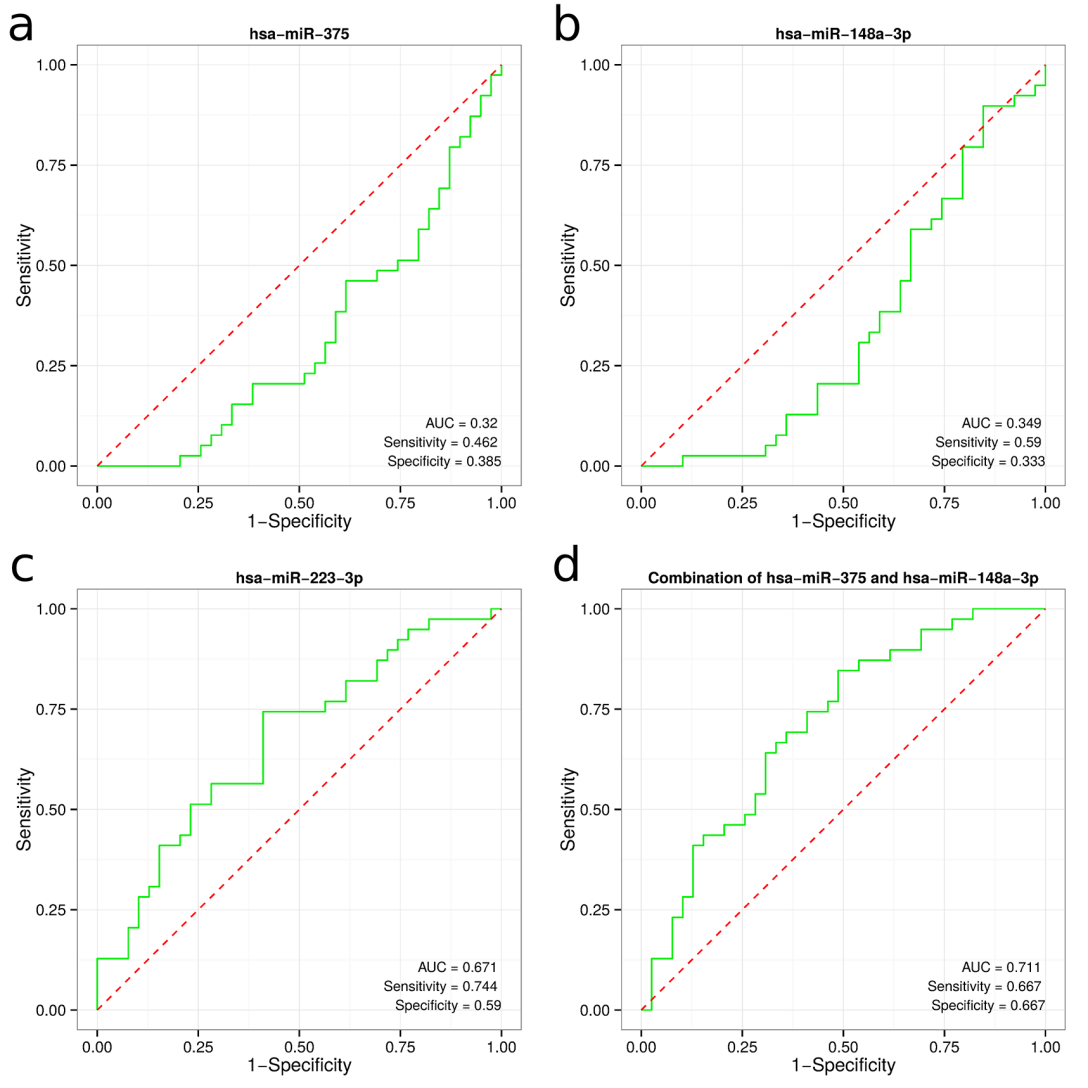
Network of candidate miRNAs and their putative target genes. Network includes the individual miRNAs (red circles) and four types of their predicted mRNA target genes (hexagons), obtained from miRTarBase and miRecords databases. The pink color represents target genes which are regulated by a single miRNA. The orange and green colors indicate target genes regulated simultaneously by two or three distinct miRNAs, respectively. GC-associated target genes retrieved from DisGeNet database are represented by blue hexagons. The databases included in the regulatory interaction networks are identified by the color of the connecting arrows: miRTarBase (blue) and miRecords (red).

In GC tissue validated miR-148a-3p, miR-204-5p, miR-223-3p and miR-375 were selected for further analysis in plasma samples. The relative levels of the candidate mRNAs in individual samples were determined using qRT-PCR (**Fig 3**). The miR-16-5p was used as the endogenous control to normalize the expression levels of candidate miRNAs. To date, the discussion over selection of most appropriate reference genes in miRNA profiling studies is ongoing. Traditionally used small nuclear RNAs (RNU6A, RNU44, RNU48 and others) might be unstable in plasma and can introduce bias in the experiment. We have performed a thorough literature analysis, before choosing miR-16-5p as a reference gene. Previous studies have identified miR-16-5p as an endogenous control miRNA, which might serve as an accurate reference gene due to its relative stable expression level in serum [37][21]. The expression levels of miR-148a-3p and miR-375 showed significant down-regulation in GC plasma; while miR-223-3p was significantly up-regulated (FDR adjusted p < 0.05). No significant differences were detected in the expression levels of miR-204-5p (**Fig 3**). To investigate the characteristics of differentially expressed miRNAs as potential biomarkers of GC, the ROC curve analysis was performed. The ROC curves of miR-148a-3p, miR-375 and miR-223-3p showed AUC value of 0.349 (95% CI, 0.289–0.408), 0.32 (95% CI, 0.261–0.377) and 0.671 (95% CI, 0.614–0.731), respectively (**Fig 4**). In the univariate gene expression average analysis of the down-regulated miR-148a-3p and miR-375, the resulting ROC curve had an AUC value of 0.711 (95% CI 0.657–0.769), which corresponds to moderate separation between the GC and control samples (**Fig 4**). Specificities and sensitivities of candidate miRNAs are shown in **Fig 4**.

### Target gene prediction

**Fig 5:**
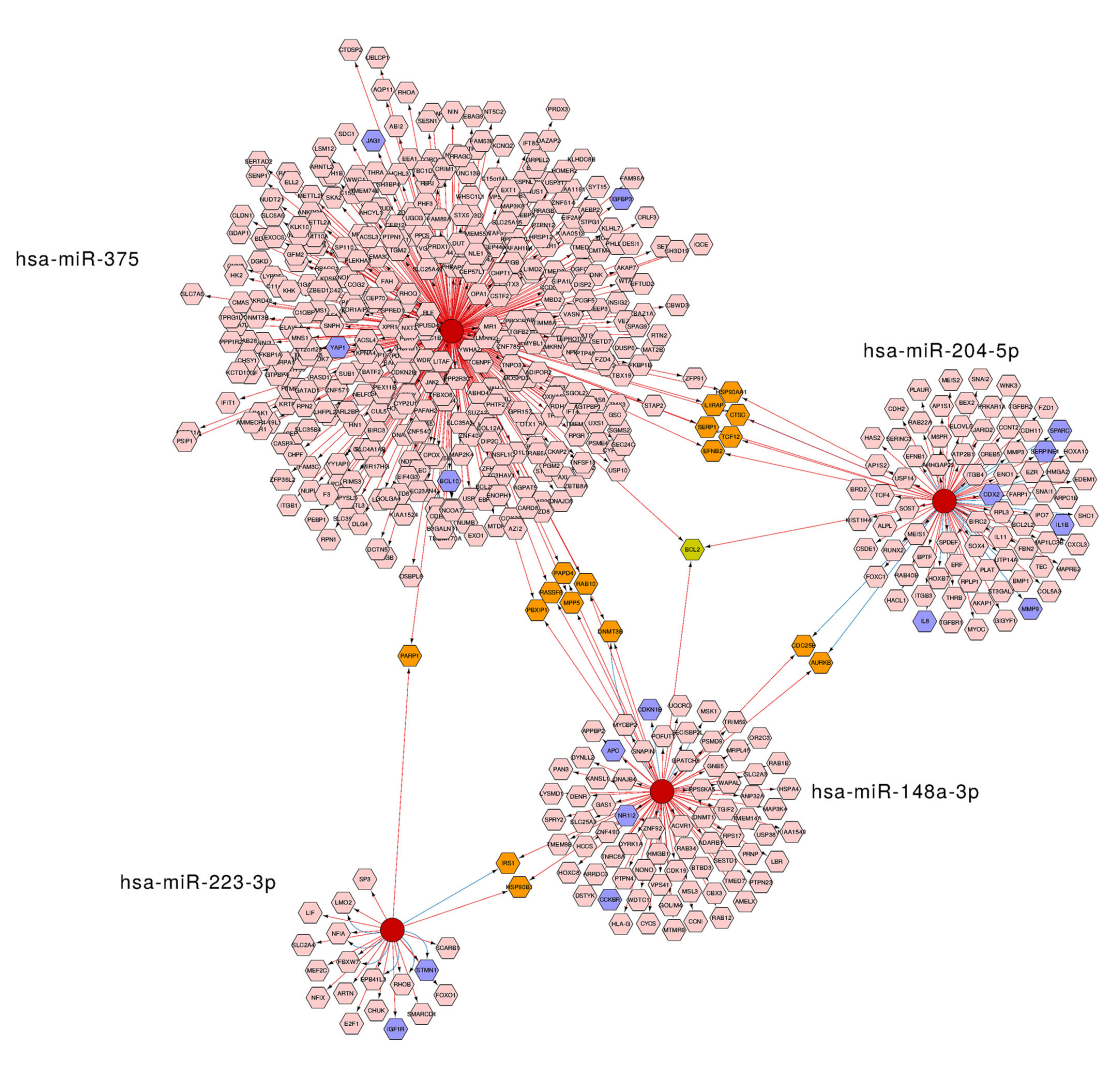
Expression levels of *BCL2* and *DNMT3B* in GC tissue and correlation analysis with their putatively targeting miRNAs. (**a**) Expression levels of *BCL2* and *DNMT3B* was analyzed using qRT-PCR. The data are represented as log2 2-(deltaCt) values; Pearson correlation analysis: (**b**) between relative expression levels of *DNMT3B* and relative expression levels miR-375; (**c**) between relative expression levels of *DNMT3B* and relative expression levels miR-148a-3p; (**d**) between relative expression levels of *BCL2* and relative expression levels miR-148a-3p; (**e**) between relative expression levels of *BCL2* and relative expression levels miR-204-5p; (**f**) between relative expression levels of *BCL2* and relative expression levels miR-375 in gastric tissue samples. P value below 0.05 was considered significant.

To further investigate the possible targets of miR-148a-3p, miR-204-5p, miR-223-3p and miR-375, all of their experimentally validated miRNA-target interactions were obtained from two different databases (miRTarbase and miRecords). The data was visualized in Cytoscape as a biological network containing all of the mentioned miRNAs and their target interactions (**Fig 5**). Each interaction in the network consisted of two nodes, a regulatory miRNA node (red) and a target mRNA node (pink) connected through one directed edge. Overall, the network included 593 validated targets, 419 of them assigned to miR-375, 88 assigned to miR-204-5p, 83 assigned to miR-148a-3p and 21 assigned to miR-223-3p. The network analysis revealed that miR-148a-3p and miR-375 had six shared targets (MPP5, DNMT3B, PAPD4, RASSF8, PBXIP1 and RAB10), miR-204-5p and miR-375 also had six shared targets (SERP1, CTSC, EFNB2, HSP90AA1, TCF12 and IL1RAP), miR-148a-3p and miR-204-5p had two shared targets (AURKB and CDC25B), miR-223-3p and miR-148a-3p also had two shared targets (HSP90B1 and IRS1), miR-223-3p and miR-375 had one shared target (PARP1) and miR-148a-3p, miR-204-5p and miR-375 also had one shared target (BCL2). Target genes which were regulated simultaneously by two or three miRNAs are represented in orange and green colors, respectively (**Fig 5**).

### Disease-associated gene enrichment analysis

In order to identify whether the disease-associated genes were significantly enriched in our set of miRNA targets, enrichment analysis was performed. First, the gene list was retrieved from DisGeNet database. As query for the database we used the term “gastric adenocarcinoma” (umls: C0149826). After filtering out miRNA, lncRNA and genes which were not in miRTarbase and miRecords, 115 genes were identified to be associated with GC. In the network analysis 20 of GC-associated genes were overlapped, 4 (BCL10, YAP1, IGFBP3 and JAG1) of them assigned to miR-375, 6 (IL8, MMP9, IL1B, CDX2, SERPINE1 and SPARC) assigned to miR-204-5p, 4 (CDKN1B, APC, NR1I2 and CCKBR) assigned to miR-148a-3p and 2 (STMN1 and IGF1R) assigned to miR-223-3p. Target genes which were associated with GC are represented in blue (**Fig 5**). Finally, disease-associated gene enrichment analysis was performed using a hypergeometric distribution-based test which led to assess whether the number of GC-associated genes is larger than expected in the set of target genes of selected miRNAs. Enrichment analysis revealed that GC-associated genes were significantly over-represented (p < 0.05) in miR-148a-3p, miR-204-5p and miR-223-3p, while no significant enrichment was observed in miR-375 target genes (p >0.05) (**Table 3**).

**Table 3:** Enrichment analysis of GC-associated genes in miR-148a-3p, miR-204-5p, miR-223-3p and miR-375 target genes by using hypergeometrical distribution.

miRNA	Validated targets of miRNA	GC-related target genes of miRNA	GC-unrelated targets of miRNA	Number of GC related genes	Number of GC unrelated genes	p-value
miR-148a-3p	83	6	77	115	12210	1.16E-04
miR-204-5p	88	4	84	115	12210	0.0103796
miR-223-3p	19	2	17	115	12210	0.0089086
miR-375	419	4	415	115	12210	0.5452276

GC–gastric cancer.

### *BCL2* and *DNMT3B* over-expression and inverse correlation with targeting miRNAs in gastric cancer tissues

**Fig 6:**
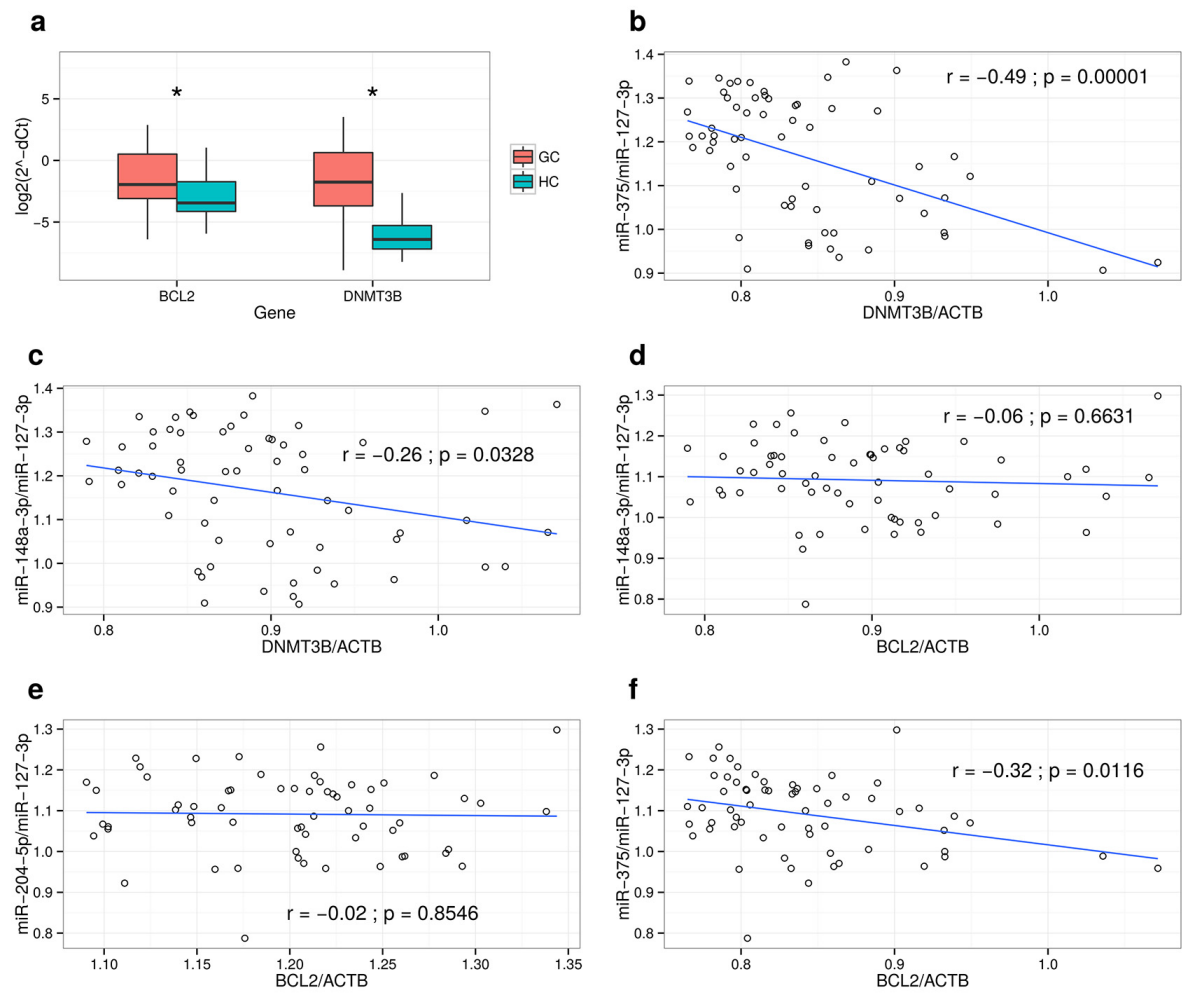
Receiver operating characteristic (ROC) curves of differentially expressed miRNAs in plasma between GC patients and healthy controls. ROC curves of miR-375 (**a**); miR-148a-3p (**b**); and miR-223 (**c**); the combination of miR-375 and miR-148a-3p (**d**).

The bioinformatical screening of potential miRNA targets identified *BCL2* as a putative target for miR-148a-3p, miR-204-5p and miR-375 and *DNMT3B* for miR-148a-3p and miR-375. All of these miRNAs in our study were found to be down-regulated in gastric cancer tissue. To further support these results, first the expression analysis of *BCL2* and *DNMT3B* genes was performed. The data of qRT-PCR showed significant increase in expression levels of both *BCL2* and *DNMT3B* genes in gastric cancer tissue. *BCL2* showed a 2.7-fold increase, while *DNMT3B* had a 16.7-fold increase in mRNA levels (**Fig 6**). Pearson’s correlation analysis was performed in order to unveil the relationship among the *BCL2* and *DNMT3B* mRNAs and their targeting miRNA levels in normal and cancerous gastric tissues (**Fig 6**). An inverse correlation was observed for both of the predicted *DNMT3B*-targeting miRNAs: miR-375 (r = −0.49; p = 0.00001) and miR-148a-3p (r = −0.26; p = 0.0328). A negative correlation was observed between *BCL2* and miR-375 (r = −0.32; p = 0.0116), whereas no relationship was found between *BCL2* and miR-148a-3p (r = −0.06; p = 0.6631) or *BCL2* and miR-204-5p (r = −0.02; p = 0.8546).

## Discussion

Altered miRNA expression profiles have been reported in GC tissue and blood samples [13][20][14][38]. Some of these deregulated miRNAs have been linked with survival, metastatic behavior of tumor and other clinicopathological features of GC [39][40]. Furthermore, miRNAs have been shown to regulate tumor growth by affecting proliferation, adhesion, invasion and migration in GC cell lines [41]. More importantly, target genes of deregulated miRNAs in GC are involved in apoptosis, cell cycle and other crucial carcinogenesis pathways [12]. Therefore, investigation of miRNAs and their potential target genes in GC may serve for further development of important diagnostic and treatment alternatives. It is evident that miRNAs are major players in gastric carcinogenesis, but we still lack precise data about the mechanisms and potential clinical applications of these molecules in GC.

Our present study aimed to determine the profile of deregulated miRNAs in GC tissues, assess them in plasma samples and to evaluate potential target genes of deregulated miRNAs. Using TaqMan miRNA TLDA cards, encompassing 377 miRNAs primers, we found 15 differentially expressed miRNAs between cancerous GC and healthy gastric tissues. Eight miRNAs were down-regulated (let-7a-5p, let-7g-5p, miR-26b-5p, miR-30b-5p, miR-135a-5p, miR-148a-3p, miR-204-5p, miR-375), while seven miRNAs were up-regulated (miR-146b-5p, miR-155-5p, miR-214-3p, miR-223-3p, miR-224-5p, miR-331-3p and miR-484). Our profiling results did not reveal some of the commonly deregulated miRNAs, which have been previously reported to be deregulated in other studies, including miR-21-5p, miR-25-3p, miR-92a-3p, miR-29c-3p, and some others [15][19][14]. One of the possible explanations for these missing miRNAs might be linked with very strict statistical criteria which were applied for TLDA analysis of our data (FDR adjusted p-value <0.01 and fold change >2). Furthermore, some level of discrepancy in miRNA profiling results among the studies might result from differences in anatomical location of the stomach, histological subtype, statistical analysis and especially profiling methods [18]. A study by Mestdagh et al. shows that different platforms of miRNA profiling have different detection rates, specificity and reproducibility, and that the most suitable platform should be chosen on the basis of the experimental setting and the specific research questions (Mestdagh et al., 2014).

In the second stage of our study we used qRT-PCR in replication cohort including six most deregulated miRNAs from the profiling phase (miR-135a-5p, miR-148a-3p, miR-155-5p, miR-204-5p, miR-223-3p and miR-375). We showed that miR-148a-3p, miR-204-5p, miR-223-3p and miR-375 were consistently deregulated between normal and cancerous GC tissues. The results of our replication cohort are supported by previous reports. Several studies have shown that miR-148a-3p was significantly down-regulated in GC cell lines and tissue samples compared to the adjacent normal tissues [42][43]. Low expression levels of miR-375 were also reported by studies, hypothesizing that miR-375 was associated with gastric carcinogenesis [44][16]. We identified that miR-223-3p was significantly up-regulated in GC tissue compared to normal tissue, as suggested by several previous reports [15][19]. MiR-204-5p has been less frequently reported in GC miRNA profiling studies; nevertheless, our results are in line with a previous study which showed significant down-regulation of this miRNA in GC tissue and GC cell lines [45]. MiR-135a-5p expression was lower in GC tissues than in control subjects; however, the observed down-regulation trend was similar to previous reports [15] and might have reached required significance with a higher number of individuals within the groups. It is worth pointing out that we did not find differences for miR-155-5p in our replication cohort comparing control subjects and GC patients. Most likely this finding is linked with the fact that, in the initial profiling cohort, control subjects were *H*. *pylori* free, while in the replication set, some of the control subjects had positive serology for this bacterium. It is well known that miR-155-5p is involved in inflammatory pathways including *H*. *pylori* gastritis [46]; therefore, this might give a certain bias to our results. Our results are in line with previous study by Link et al. (2015), where the difference in the expression of miR-155 in biopsies from GC compared to controls did not reach statistical significance [18]. In the replication stage of our miRNA profiling study we selected only six miRNAs, which showed the highest biological relevance and statistical significance. We agree that it would be worthwhile to look at all significantly deregulated miRNAs and that could be a potential task in further studies.

To investigate the potential role of differentially expressed miRNAs as biomarkers in GC, we analyzed deregulated miRNAs in plasma samples. Circulating miRNAs, particularly the combination of multiple miRNAs, may present as promising biomarkers for the diagnosis of gastrointestinal cancers [21]. A study by Zhu et al. demonstrated that a panel of five miRNAs may serve as a potential biomarker in detecting early stage GC [47]. To date, reported miRNA profiles in GC serum or plasma differ considerably among separate studies [21]. We found that expression levels of miR-148a-3p and miR-375 were down-regulated in GC plasma; while miR-223-3p was significantly up-regulated. Individual analysis of ROC curves for miR-148a-3p, miR-375 and miR-223-3p expression in plasma samples suggests poor diagnostic performance for GC and these results are in line with certain previous studies [21]. However, when combining two down-regulated miRNAs (miR-148a-3p and miR-375), we obtained an AUC value of 0.71, which corresponds to moderate separation between the GC and control samples. Based on our findings, these circulating miRNAs have relatively weak diagnostic value and may not be applicable as sole biomarkers for GC. We could postulate that with increasing number of miRNAs analyzed in the plasma or in adjunction with other cancer related biomarkers, higher diagnostic accuracy might be achieved, but these speculations need further research.

In the third stage of our study, to gain further insight into the pathogenic role in gastric cancerogenesis of miR-148a-3p, miR-204-5p, miR-223-3p and miR-375, we obtained miRNA validated target genes and performed disease-associated gene enrichment analysis. MiR-148a-3p, miR-204-5p and miR-223-3p, which were significantly enriched in GC-associated target genes, are clearly implicated in GC-related pathways. MiR-148a-3p, down-regulated in GC, was shown to influence tumor cell growth, migration, adhesion, invasion and angiogenesis in GC by targeting CDKN1B (p27), NR1I2 (PAR2) and CCKBR genes [42][48]. MiR-204-5p may act as a tumor-suppressor by targeting IL-8, SOX4, USP47 and RAB22A genes and regulating the apoptosis, proliferation, invasion and tumor progression in GC [49][45]. Important studies have shown that miR-223-3p, up-regulated in GC, could directly target STMN1and IGF1R genes, resulting in significantly suppressed proliferation, growth rate and colony formation *in vitro* and *in vivo* [50][51]. In our disease-associated target gene enrichment analysis, miR-375 was not significantly enriched in GC-associated target genes. However, a study by Shen et al. showed that miR-375 overexpression suppressed the proliferation of human gastric cancer cells *in vitro* [52]. Another study by Xu et al. showed that miR-375 may be negatively regulated by Snail and involved in gastric cancer cell migration and invasion, potentially by targeting JAK2 [53].

Our network analysis revealed that potential targets of these miRNAs include important cancer-related genes which are regulated simultaneously by two or three distinct miRNAs. For example, miR-148a-3p, miR-204-5p and miR-375, found to be down-regulated in GC, simultaneously target *BCL2* oncogene, which acts as an anti-apoptotic factor and regulates apoptosis (Zhang et al., 2011). A study by Sacconi *et al*. showed that *BCL2* gene is an important component of the complex molecular network underlying poor response of gastric tumors to anticancer treatment and can be used as independent prognostic factor for GC patients [54]. *DNMT3B* is an oncogene which was found to be targeted by miR-375 and miR-148a-3p. It was revealed that *DNMT3B* gene is responsible for de novo methylation during embryogenesis and promotes tumor genesis of gastric cancer [55][38]. To support the observations revealed by our bioninformatical analysis, we evaluated the expression of *BCL2* and *DNMT3B* in GC tissues and found that both of them are up-regulated. Furthermore, we showed a link between expression of target genes and targeting miRNAs—significant correlation was determined for both of the predicted *DNMT3B*-targeting miRNAs (miR-375 and miR-148a-3p) and for one of *BCL2* targeting miRNAs (miR-375). It is well known that in certain cases, predicted target genes of miRNAs do not always correlate with real time alterations in the tissue [56]. Nevertheless, it is very likely that the relationships between miRNAs and their targets are not one-to-one but multiple-to-multiple in GCs, and that these complex relationships may be related to gastric carcinogenesis [57]. Investigation of other potential target genes identified by our bioinformatical tools and their validation by additional functional analysis in cell lines would be a valuable target in further research studies.

Our study was underpowered to determine pattern of miRNA expression among different anatomical and histological subtypes of GC due to a relatively small number of individuals within the groups. Therefore, larger scale studies with high numbers of individuals stratified by different clinical and pathological features of GC are further needed to outline potential differences among the subgroups of GC patients. There are some limitations to our disease-associated target gene enrichment analysis, possibly related to the incompleteness of the DisGeNET database due to inaccuracies derived from text-mining. As a result of this, not all of the GC-associated genes could have been included in our set of genes. Moreover, in our analysis we used all miRNA-target interactions retrieved from miRecords and miRTarbase databases, including weak evidence interactions based on microarray method, which could have caused some false-positive or indirect miRNA-target interactions.

## Conclusions

Our study revealed miRNA profile in GC tissues and showed that miR-148a-3p, miR-223-3p and miR-375 are deregulated in GC plasma samples, but these circulating miRNAs showed relatively weak diagnostic performance as sole biomarkers. Target gene analysis demonstrated that *BCL2* and *DNMT3B* expression in GC tissue correlated with their targeting miRNA expression.

## Supporting Information

S1 Table Raw data of miRNA profiling with Taqman low density array in gastric cancer patients and healthy controls. (XLS)

S2 Table Raw data of miR-135a-5p, miR-148a-3p, miR-155-5p, miR-204-5p, miR-223-3p and miR-375 expression in tissues of gastric cancer patients and healthy controls. (XLSX)

S3 Table Raw data of miR-148a-3p, miR-204-5p, miR-223-3p and miR-375 expression in plasma samples of gastric cancer patients and healthy controls. (XLSX)

S4 Table Raw data of BCL2 and DNMT3B expression in tissues of gastric cancer patients and healthy controls. (XLSX)
