# Correction: Analysis of Deregulated microRNAs and Their Target Genes in Gastric Cancer

**DOI:** 10.1371/journal.pone.0135762

**Published:** 2015-08-12

**Authors:** Simonas Juzėnas, Violeta Saltenienė, Juozas Kupcinskas, Alexander Link, Gediminas Kiudelis, Laimas Jonaitis, Sonata Jarmalaite, Limas Kupcinskas, Peter Malfertheiner, Jurgita Skieceviciene

The order of figure legends for Figs 4, 5 and 6 are switched in the published article. Please view Figs 4, 5 and 6 with their correct figure legends here.

**Fig 4:**
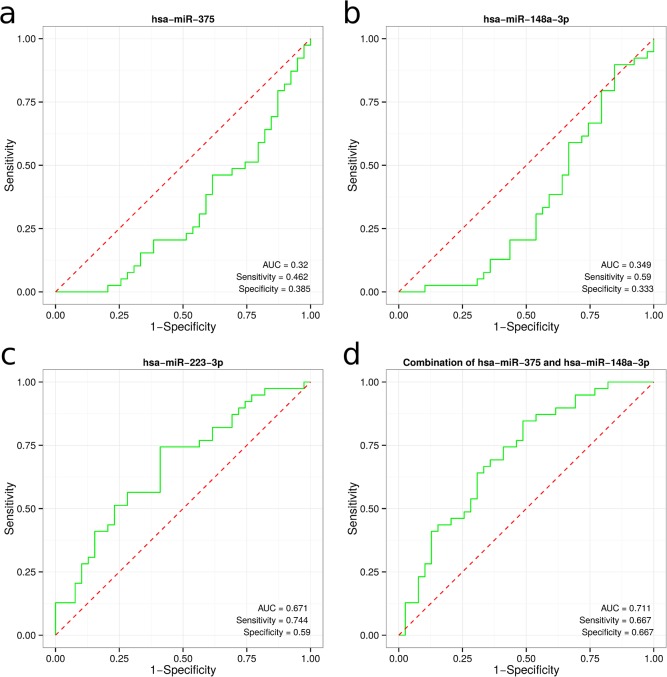
Receiver operating characteristic (ROC) curves of differentially expressed miRNAs in plasma between GC patients and healthy controls. ROC curves of miR-375 (a), miR-148a-3p (b) and miR-223 (c). The combination of miR-375 and miR-148a-3p (d).

**Fig 5:**
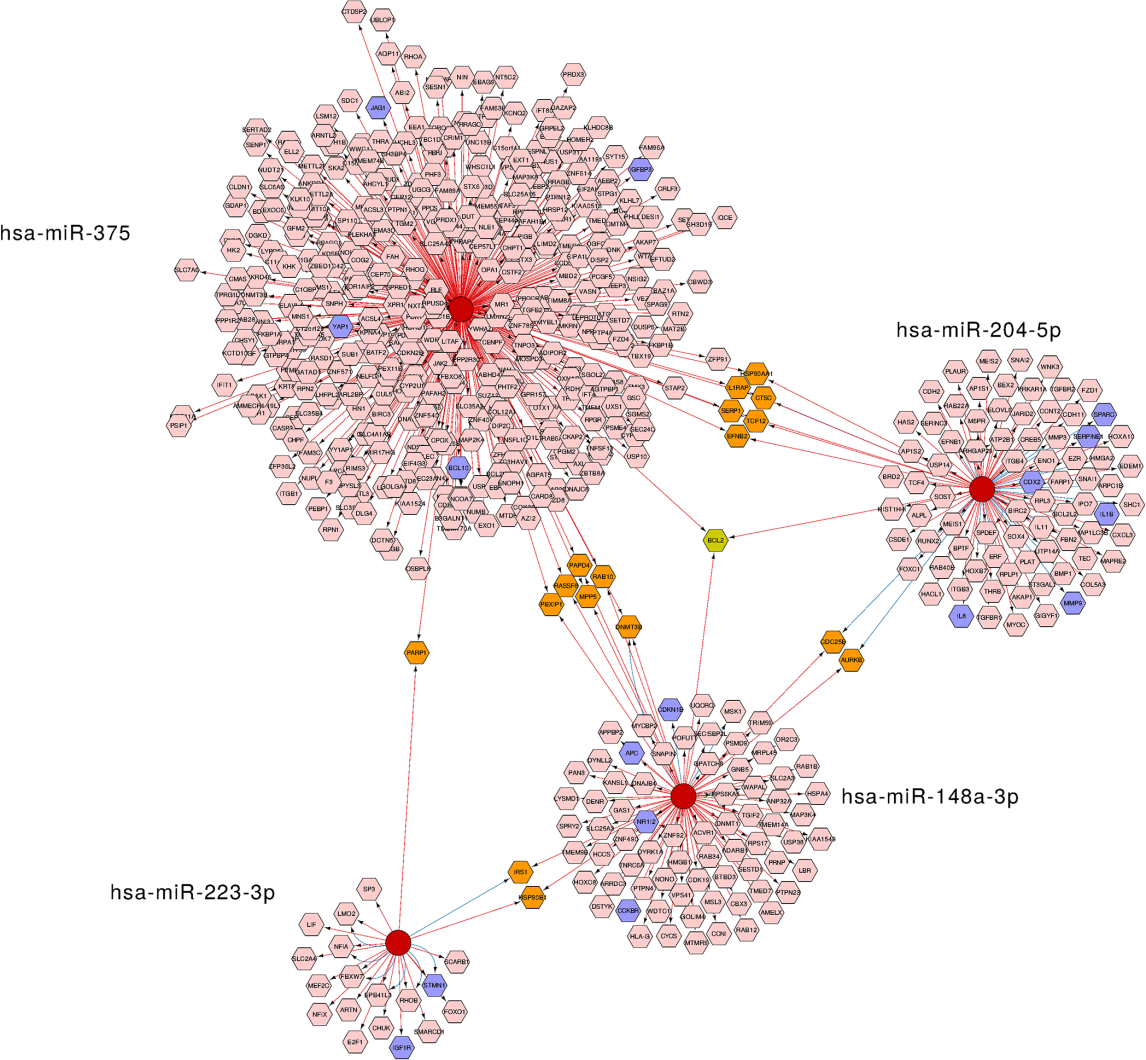
Network of candidate miRNAs and their putative target genes. Network includes the individual miRNAs (red circles) and four types of their predicted mRNA target genes (hexagons), obtained from miRTarBase and miRecords databases. The pink color represents target genes which are regulated by a single miRNA. The orange and green colors indicate target genes regulated simultaneously by two or three distinct miRNAs, respectively. GC-associated target genes retrieved from DisGeNet database are represented by blue hexagons. The databases included in the regulatory interaction networks are identified by the color of the connecting arrows: miRTarBase (blue) and miRecords (red).

**Fig 6:**
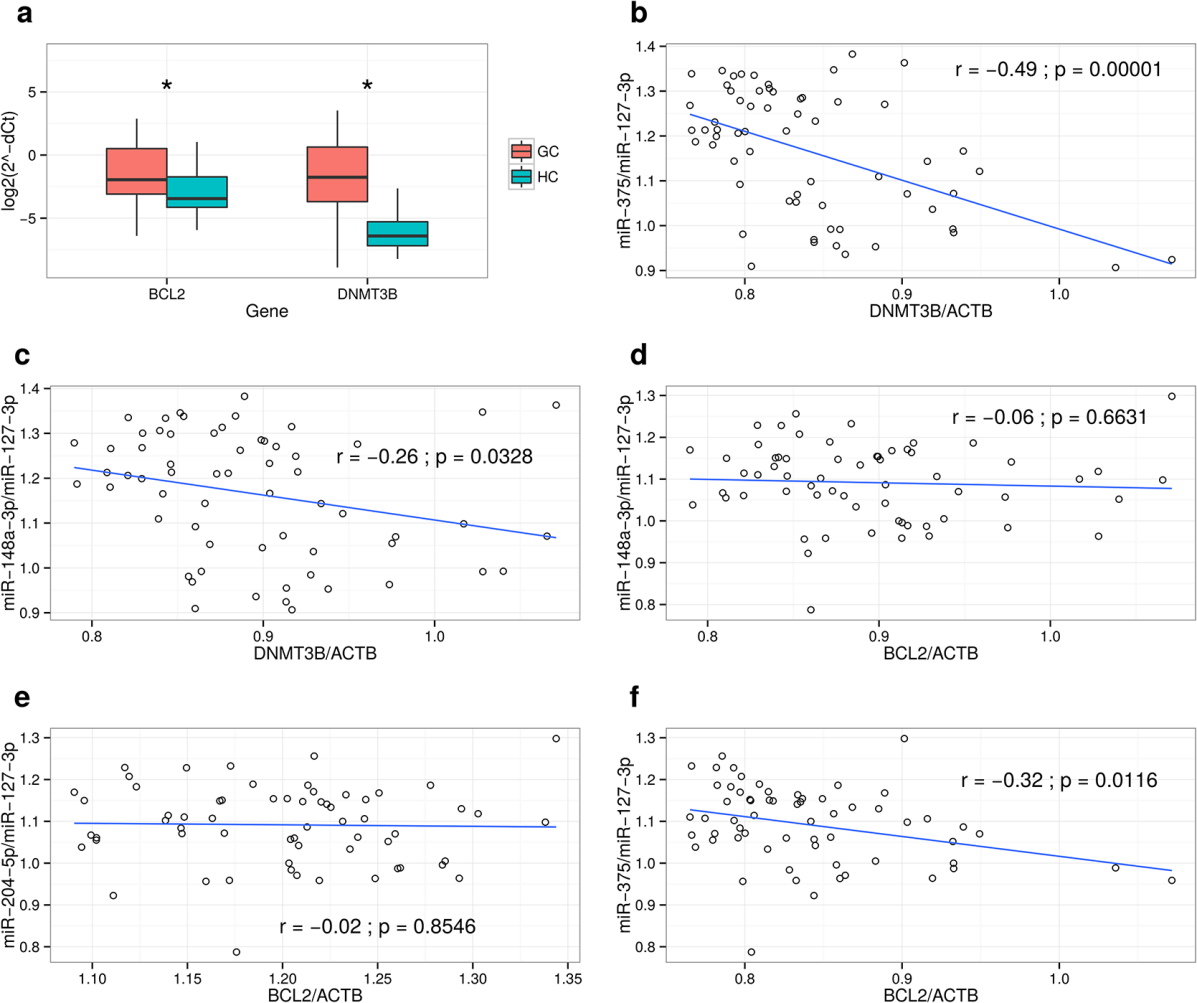
Expression levels of BCL2 and DNMT3B in GC tissue and correlation analysis with their putatively targeting miRNAs. (a) Expression levels of BCL2 and DNMT3B was analyzed using qRT-PCR. The data are represented as log2 2-(deltaCt) values. (b) Pearson correlation analysis between relative expression levels of DNMT3B and relative expression levels miR-375, (c) between relative expression levels of DNMT3B and relative expression levels miR-148a-3p, (d) between relative expression levels of BCL2 and relative expression levels miR-148a-3p, (e) between relative expression levels of BCL2 and relative expression levels miR-204-5p, (f) between relative expression levels of BCL2 and relative expression levels miR-375 in gastric tissue samples. P value below 0.05 was considered significant.
